# The effect of two calcium phosphate‐containing agents on the enamel resistance of permanent molars to demineralization: An experimental study

**DOI:** 10.1002/cre2.649

**Published:** 2022-08-22

**Authors:** Fatemeh Molaasadolah, Zohre Sadat Hosseinipour, Fatemeh Afzali, Ardavan Parhizkar, Kiana Poorzandpoush

**Affiliations:** ^1^ Department of Pediatric Dentistry, School of Dentistry Shahid Beheshti University of Medical Sciences Tehran Iran; ^2^ Department of Pediatric Dentistry, School of Dentistry AJA University of Medical Sciences Tehran Iran; ^3^ Private Practice Ashkane North Khorasan Iran; ^4^ Iranian Centre for Endodontic Research, Research Institute for Dental Sciences Shahid Beheshti University of Medical Sciences Tehran Iran; ^5^ Department of Pediatric Dentistry, School of Dentistry Shahid Beheshti University of Medical Sciences Tehran Iran

**Keywords:** calcium phosphate‐containing agents, demineralization, enamel resistance, energy‐dispersive X‐ray analysis

## Abstract

**Background:**

The main purpose of this experimental study was to determine the in vitro effects of two calcium phosphate‐containing agents (Remin Pro® and GC Tooth mousse™) on the enamel resistance of permanent molars to demineralization.

**Methods:**

Fifty extracted human third molars were randomly divided into four groups; that is the control group and three case groups treated with Remin Pro®, GC Tooth mousse™, and sodium fluoride gel. The three case groups were treated with 0.25 ml of the paste associated with each experimental group for 5 min, kept in fluoride‐free artificial saliva, and incubated at 37°C for 28 days. After the treatment regimen, 10 samples of each case group were subjected to demineralization using an acetic acid‐containing solution, and remineralization using a remineralizing solution. The morphology of enamel was observed via scanning electron microscopy and their enamel calcium/phosphorus (Ca/P) ratios were measured before/after the demineralization cycle with energy‐dispersive X‐ray analysis. Data were statistically analyzed using one‐way analysis of variance and post hoc Tukey tests.

**Results:**

The enamel Ca/P ratios in the case study groups were significantly higher than that of the control group before/after the demineralization regimen (*p* < .0001). However, the ratios were not significantly different between the case study groups after the treatment regimen and demineralization cycle (*p* > .05).

**Conclusion:**

The outcomes of the current study indicated that all three agents seemed to increase the enamel resistance of permanent molar teeth to demineralization.

## 1 BACKGROUND

Tooth decay is considered the most common oral health problem, affecting different age groups from young children to the elderly (Astasov‐Frauenhoffer & Kulik, 2021; Veeraboina et al., 2020). Due to various socioeconomic consequences of dental caries, prevention is regarded as the best way to avoid subsequent complications. Nowadays, with the introduction of noninvasive methods for noncavitated carious lesions as well as decays extending up to the dentin‐enamel junction, it has become possible to prevent/reduce the process of demineralization and caries progression (Holmgren et al., 2014). The use of fluoride and calcium phosphates has been recently taken into account as successful preventive methods in the combat against tooth decay (Veeraboina et al., 2020).

Topical fluorides have proved to exhibit remineralization effects on initial carious lesions, which may be improved through the addition of calcium and/or phosphate ions (Amaechi et al., 2019). Therefore, the use of calcium phosphate‐containing agents has been recently introduced as a preventive method, for example, the application of calcium phosphate powders/chewing gums/varnishes, specifically and casein phosphopeptide amorphous calcium phosphate complexes (CPP–ACP, e.g., GC tooth mousse™) (Meyer‐Lueckel et al., 2015) and Remin Pro® (Nourolahian et al., 2021). Different studies on the application of CPP‐ACP, that is, GC tooth mousse™ in the current study, have shown to cause an increase in calcium/phosphorus (Ca/P) ratio in tooth enamel (Mettu et al., 2015), slow progress of caries in initial lesions (Llena et al., 2009), and potential enamel remineralization capability (Pirca et al., 2019). Moreover, CPP‐ACP and fluoride have demonstrated a possible synergistic effect against caries associated with an increased remineralization ability in superficial carious lesions (Bayrak et al., 2017). It has been indicated that GC tooth mousse™ can be used as a topical crème to manage cervical dentin sensitivity (Walsh, 2010) and has shown to have a crucial role in the improvement of oral health in children who are at high risk of caries (Kilic & Gurbuz, 2021). GC tooth mousse™ contains xylitol and casein phosphopeptide amorphous calcium phosphate, and thus, can be applied for the prevention of initial enamel lesions (Cochrane & Reynolds, 2012). Remin Pro® contains hydroxyapatite, fluoride (1450 ppm), and xylitol. As a water‐based remineralizing agent, it increases the penetration of hydroxyapatite as well as fluoride into any porosity on the surface of the enamel and initial carious lesions (Ahrari et al., 2018; Ebrahimi et al., 2017), causing a smooth enamel surface and production of fluorapatite as a more stable and acid‐resistant structure compared to hydroxyapatite (Nourolahian et al., 2021). Remin Pro® is believed to prevent erosion as well as the demineralization of teeth (Pallepati & Yavagal, 2020), and assist in neutralizing acids in dental plaque (Esfahani et al., 2015). Therefore, this study focused on using Remin Pro® and GC tooth mousse™ due to (i) their remineralization capability and (ii) materials' applications in preventive dentistry.

Scanning electron microscopy (SEM) as one of the most sensitive techniques in the evaluation/observance of the surface and/or structure of a material can be used for the morphological examination of the enamel surface and assessment of the remineralization/demineralization processes of dental caries (Kamath et al., 2017). Additionally, energy‐dispersive X‐ray analysis (EDXA) is applied for the chemical characterization of a material; for example, the determination of the Ca/P ratio of a constituent including enamel (Parhizkar et al., 2020).

The present in vitro study aimed to investigate the effects of two calcium phosphate‐containing agents (Remin Pro® and GC tooth mousse™) and MasterDent fluoride gel on the enamel resistance to demineralization of permanent molars using SEM‐EDXA.

## 2 METHODS

The current in vitro study was approved by the “Ethics Committee, Vice Chancellor for Research, School of Dentistry, Shahid Beheshti University of Medical Sciences (IR.SBMU.RIDS. REC.1396.597).”

Fifty hopeless sound third molar teeth were extracted and polished with fluoride‐free pumice paste and distilled water using a low‐speed handpiece and rubber cup for 10 s, followed by copious irrigation with deionized water for 30 s. Then, the teeth were cut with a diamond disc bur, at the level of the cementoenamel junction. Next, tooth crown pieces were sectioned to buccal or lingual portions with dimensions of 2 × 4 mm, and 50 sound enamel specimens were randomly selected. Then, the inner side (dentinal aspect) of specimens was completely flattened using a fissure bur. Afterward, the specimens were divided into four groups; that is, the control group (*n* = 5, untreated specimens) and three case groups treated with Remin Pro® (*n* = 15) (VOCO GmbH, Germany), GC Tooth mousse™ (*n* = 15) (GC, Japan), and sodium fluoride gel (*n*= 15) (2%, MasterDent, USA).

**Figure 1:**
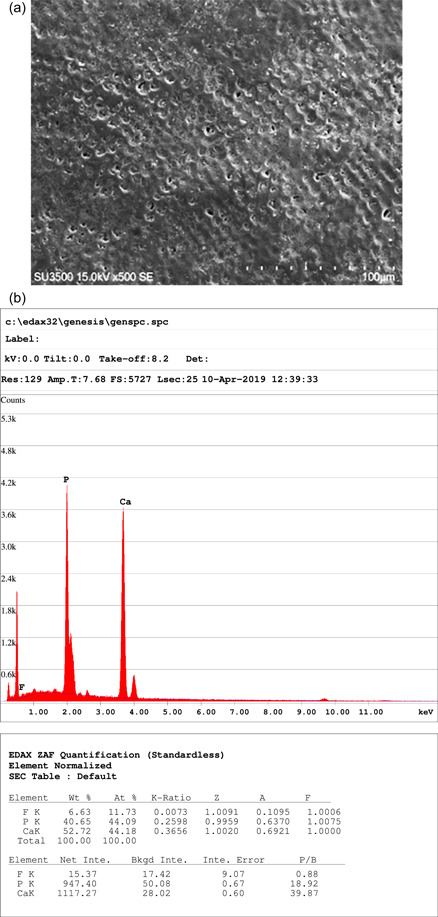
(a) Enamel surface; control group, (b) enamel surface; control group, energy‐dispersive X‐ray analysis showing the weight percentages of calcium and phosphorus

The specimens in the control group underwent no treatment regimen and their initial mineral content was evaluated using SEM (x500; SU3500, Hitachi, Japan) equipped with an EDXA analyzer (Octane Parime, Ametek EDXA, USA) (Figure 1a). The buccal or lingual surfaces of specimens in the three case groups were initially rinsed with deionized water and then, 0.25 ml of the corresponding paste related to each experimental group was placed on the surface of specimens with a syringe for 5 min according to the manufacturer's instructions. Subsequently, the excesses were removed from the surface of specimens using a cotton roll. Then, the specimens were kept in fluoride‐free artificial saliva and incubated at 37°C for 28 days. The mentioned procedure was performed and repeated on Days 7, 14, 21, and 28. To prevent surface changes during this period, the saliva solution was renewed twice a week. After 28 days, five specimens from each case group were randomly taken and analyzed using SEM‐EDXA.

Ten remaining specimens in each case group were subjected to a demineralization regimen (2.9 gr NaCl, 0.12 gr CaCl_2_, 0.13 gr NaH_2_PO_4_, 5 ml NaN_3_, 1.5 ml Acid acetic) for a week. The specimens were immersed in the demineralizing solution with pH=4.4 for 6 h and then, in remineralizing solution (2.9 gr NaCl, 0.12 gr CaCl_2_, 0.13 gr NaH_2_PO_4_, 5 ml NaN_3_) with pH = 6.5 for 18 h (pH Cycling). Ten samples from each group were dried, examined under SEM for their morphology, and assessed by an EDXA analyzer for their mineral content.

### 2.1 Statistical analysis

Data were analyzed using the statistical package for social sciences (SPSS) software, version 21. Differences in mineral content of the three groups of specimens were evaluated using one‐way analysis of variance (ANOVA) before and after exposure to remineralizing agents. The control group was compared with the case groups before and after exposure to demineralizing/remineralizing solutions using one‐way ANOVA test. The probability value (*p*‐value) was set at .05 in the present study.

## 3 RESULTS

**Figure 2:**
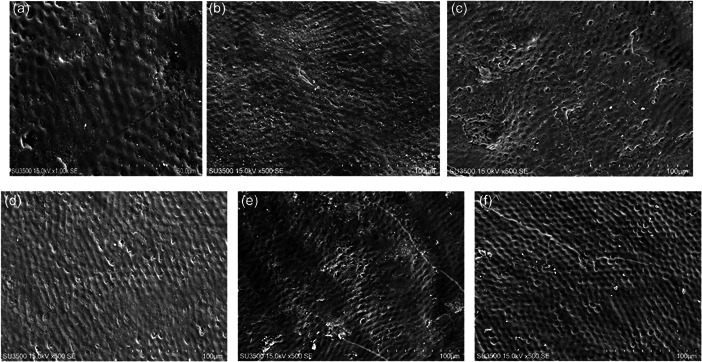
(a) Enamel treated with Remin Pro® before demineralization, (b) enamel treated with Remin Pro® after demineralization, (c) enamel treated with GC tooth mousse before demineralization, (d) enamel treated with GC tooth mousse after demineralization, (e) fluoride gel treated enamel before demineralization, (f) fluoride gel treated enamel after demineralization

SEM micrographs showed a similar covering layer on the surface of enamel prisms in all treated groups after the treatment regimen of VOCO Remin Pro®, GC Tooth Mousse™, and Fluoride Gel Masterdent despite exhibiting no signs of the layer before the treatment in all groups. Furthermore, a comparable number of porosities in enamel prisms before and after pH cycle was revealed by SEM (Figure 2a–f). Additionally, enamel prisms showed less porosity compared with the control group.

Table 1 shows the comparison between the mineral content (Ca/P ratio) in the control group and the three treated groups before and after pH cycling. The comparison indicated that the mean Ca/P ratio in the control group was significantly different from that of the three case groups (*p* < .05). However, there were insignificant differences between the case group specimens after the treatment regimen. Moreover, the specimens that underwent pH cycling showed insignificant differences before and after the cycle within their group and with the other groups (*p* > .05).

**Table 1:** The mean mineral content (calcium/phosphorus [Ca/P] ratio) in the control group and the three treated groups before and after pH cycling

	*N*	Mean	Standard deviation (±)	Standard error	95% Confidence interval		Minimum (wt %)	Maximum (wt %)
					Lower bound	Upper bound		
Control group	5	1.26	0.04	0.019	1.20	1.33	1.22	1.30
Remin Pro® *before* pH cycling	5	1.82	0.02	0.01	1.78	1.86	1.81	1.84
Remin Pro® *after* pH cycling	10	1.80	0.05	0.02	1.76	1.85	1.75	1.85
GC Tooth Mousse™ *before* pH cycling	5	1.77	0.08	0.04	1.58	1.97	1.69	1.84
GC Tooth Mousse™ *after* pH cycling	10	1.84	0.05	0.02	1.79	1.89	1.78	1.94
Fluoride Gel Masterdent *before* pH cycling	5	1.79	0.04	0.02	1.70	1.89	1.75	1.83
Fluoride Gel Masterdent *after* pH cycling	10	1.86	0.14	0.05	1.73	1.99	1.73	2.15
Total	50	1.76	0.20	0.03	1.69	1.83	1.22	2.15

The outcomes of the current study showed that the Ca/P ratio significantly increased in all three case groups after the treatment regimen; nevertheless, the difference between the case groups was not significant. However, after the demineralization cycle, the Ca/P ratio was found to be higher in case groups than that in the control group (*p* < .05) (Figures 1a,b).

Consequently, the outcomes of the current investigation showed that the Ca/P ratio was the highest in Remin Pro® (mean = 1.82) and lowest in GC tooth mousse™ (mean = 1.77) before the pH cycling and the highest in Fluoride Gel Masterdent (mean = 1.86) and the lowest in Remin Pro® (mean = 1.80) after the cycling, although the differences between groups were not significant.

## 4 DISCUSSION

The present study investigated two recently introduced calcium phosphate‐containing agents and fluoride gel as well as their effects on the resistance of permanent teeth enamel to the demineralization process, indicating an increase in Ca/P ratio in the examined specimens. Besides, the agents showed promising results on the remineralization pattern and positive effects on the enamel resistance to demineralization.

In the current study, SEM/EDXA were used to examine the surface/layer morphology and analysis of the mineral content of the affected enamel surfaces as a noninvasive measurement approach. The comparison between the lower number of porosities in the groups experimented with and that of the control group, revealed by SEM, indicated a higher possibility for an increase in enamel resistance and thus, a potential decrease in the formation of carious lesions in the groups tested. In addition, the porosities read by SEM in the groups experimented with after pH cycling showed insignificant differences with porosities before the corresponding cycling, which, in turn, exhibited the resistance of the specimens against demineralization. Moreover, SEM/EDXA showed a statistically significant increase in Ca/P ratio, indicating an appropriate matrix for possible remineralization (Hegde & Moany, 2012), which efficiently alters and increases the microhardness of the enamel (Swarup & Rao, 2012). These findings are in line with a similar study by Tahmasebi et al., who reported the stimulating effects of the remineralizing agents on enamel (Tahmasbi et al., 2019). The increase in Ca/P ratio seemed to result in the deposition/formation of a covering layer over enamel prisms, and therefore, a reduction in the number of porosities. Besides, using SEM/EDXA may be considered noninvasive in comparison to the Vickers hardness test in which an indenter is used for the exploration of microhardness of enamel (Tijana et al., 2021). Therefore, SEM/EDXA and the measurement of the Ca/P ratio may be used as an alternative to predict the remineralization capability of an agent and thus, a possible alternative to the Vickers test. However, more investigations are necessary to affirm this basic method.

Our investigation revealed that the highest Ca/P ratio was found in the specimens treated with Remin Pro®. Similarly, it has been demonstrated that the application of Remin Pro® has presented remineralizing effect on the enamel surface due to an increase in Ca/P ratio as a probable result of the existing hydroxyapatite (Cherian et al., 2020).

In the present study, Masterdent fluoride gel containing 2% sodium fluoride was used and showed an increase in the mineral content (Ca/P ratio), leading to better remineralization of the enamel. In addition, the gel could result in enhanced enamel resistance due to the production of fluorapatite (Lee et al., 2017). Additionally, it has been shown that the fluoride gel may prevent demineralization and improve remineralization through the formation of fluoride‐containing (F‐C) alkaline compounds around the enamel surface (Ebrahimi et al., 2017).

Calcium phosphate‐containing agents, as well as fluoride‐based materials, are considered relative determiners for the de/remineralization of dental structure in the oral cavity (Abou Neel et al., 2016; Diamanti et al., 2010) and have shown to cause a possible decrease in tooth susceptibility to carious lesions (Elgamily et al., 2018). The application of GC tooth mousse™, as a CPP‐ACP containing material, with its two main ions of calcium and phosphate essential for the remineralization process, showed to cause a significant increase in Ca/P ratio; nevertheless, it was slightly less than that of the other two groups. The outcomes could be due to the fact that GC Tooth mousse™ does not contain hydroxyapatite and fluoride. CPP‐ACP is responsible for the release and increase of calcium/phosphate ions, indicating a remineralizing effect. Therefore, GC Tooth mousse™ may help the remineralization process and result in an increase in the strength of enamel (Ferrazzano et al., 2011). These findings confirm the outcomes of an investigation by Bar‐Hillel et al.; they demonstrated an increase in the subsurface Ca/P ratio following the treatment of demineralized enamel with GC Tooth Mousse™ (Bar‐Hillel et al., 2012). Consequently, the current study revealed that the highest increase in Ca/P ratio and mineral content was observed in Remin Pro® (44.1%), followed by Masterdent fluoride gel (42%) and GC Tooth mousse™ (40.6%) compared to the control group. Additionally, the outcomes are in line with similar research showing higher surface resistance after the treatment with Remin Pro®, CPP–ACP agents, and sodium fluoride (Nourolahian et al., 2021; Sahiti et al., 2020; Tahmasbi et al., 2019).

Besides, our study disclosed a higher Ca/P ratio and greater mineral content in the treatment regimen with Remin Pro® compared to fluoride gel. Since Remin Pro® contains hydroxyapatite as well as fluoride ions, it is expected that fluoride gel should have less effect on the increase of the mineral content (Gavrilă et al., 2015). However, Chaudhary et al. investigated the effects of CPP‐ACP/calcium sodium phosphosilicate on remineralization of demineralized enamel in comparison with fluoride‐containing materials and showed that CPP‐ACP agents increased the mineral content of enamel far more than F‐C compounds (Chaudhary et al., 2017). Nonetheless, the present study indicated a higher increase in mineral content using sodium fluoride than GC tooth mouse™ (CPP‐ACP agent). This inconsistency may be due to the difference in methodology since the treatment regimen in the current study was applied to enamel surfaces before demineralization, whereas Chaudhary used the regimen after the demineralization of the enamel surface. However, further research and well‐designed randomized clinical trials should be conducted for the possible application of the aforementioned materials in dental treatments.

A limitation to the present experimental in vitro study was that despite efforts to the establishment of creating a comparable environment to the oral cavity, differences (e.g. saliva flow, constant dilution of ions, such as fluoride, calcium, and phosphate, and regular rinse) were not considered.

## 5 CONCLUSIONS

Within the natural limitations of an experimental study:

1. The current investigation showed that Remin Pro®, GC Tooth mousse™, and sodium fluoride gel were able to increase the Ca/P ratio in the enamel samples.
2. The application of Remin Pro®, GC Tooth mousse™, and sodium fluoride gel could enhance the enamel resistance to demineralization.
3. The application of Remin Pro®, GC Tooth mousse™, and sodium fluoride gel should help clinicians promote enamel resistance and thus, lower the possibility of carious lesions.

## AUTHOR CONTRIBUTIONS

**Fatemeh Molaasadolah**: Conceptualization; data/formal analysis; investigation; methodology; project administration; supervision; validation; visualization; writing—original draft; and writing—review/editing. **Zohre Sadat Hosseinipour**: Conceptualization; investigation; methodology; validation; visualization; and writing—original draft. **Fatemeh Afzali**: Conceptualization; data/formal analysis; investigation; methodology; project administration; and visualization. **Ardavan Parhizkar**: Conceptualization; investigation; methodology; validation; visualization; writing—original draft; and writing—review/editing. **Kiana Poorzandpoush**: Conceptualization; data/formal analysis; investigation; methodology; project administration; supervision; validation; visualization; writing—original draft; and writing—review/editing.

## CONFLICT OF INTEREST

The authors declare no conflict of interest.

## Data Availability

The data used to support the findings of the current study are available upon request from the corresponding author.
